# The role of Shunaoxin pills in the treatment of chronic cerebral hypoperfusion and its main pharmacodynamic components

**DOI:** 10.1515/med-2022-0607

**Published:** 2022-11-23

**Authors:** Jin Zhang, Nianwei Chang, Jiani Liu, Wenjuan Liu, Gang Bai

**Affiliations:** State Key Laboratory of Medicinal Chemical Biology, College of Pharmacy and Tianjin Key Laboratory of Molecular Drug Research, Nankai University, Tianjin, 300353, China; College of Traditional Chinese Medicine, Tianjin University of Traditional Chinese Medicine, Tianjin, 300193, China

**Keywords:** Shunaoxin pills, active ingredients discovery, Ca^2+^, ligustilide, senkyunolide I, chronic cerebral hypoperfusion

## Abstract

Chronic cerebral hypoperfusion (CCH) is a frequent ischemic cerebrovascular disease that induces brain dysfunction. Shunaoxin pills (SNX) are traditional Chinese medicines (TCM), frequently used for the treatment of CCH. The purpose of this study was to develop an activity-based screening system to identify the active ingredients of SNX. We developed a model of CCH and revealed that SNX induces cerebrovascular dilatation and protects against CCH-induced nerve cell injury in rats. Using the transcriptome analysis, we found that Ca^2+^-related signaling pathways play a major role in the effect of SNX against CCH. We developed an activity-based screening system based on the ultra-high performance liquid chromatography with quadrupole time-of-flight mass spectrometry coupled with a dual-luciferase reporter calcium assay to identify the active components of SNX. As a result, SNX dilates cerebral blood vessels, increasing cerebral blood flow by modulating calcium-related signaling pathways and regulating calcium homeostasis. Two calcium antagonists, ligustilide and senkyunolide I, were identified as active ingredients in SNX. In conclusion, we developed a rapid screening method suitable for the discovery of active natural products in TCM by integrating genomics and target pathway-oriented spectroscopic analysis.

## 1 Introduction

Chronic cerebral hypoperfusion (CCH) is a frequent clinical ischemic cerebrovascular disease that is closely related to the occurrence and development of vascular dementia and Alzheimer’s disease [1,2]. Using calcium antagonists (CAA) to improve cerebral circulation represents the first-line treatment of CCH [3], as they have higher efficacy and fewer side effects than other therapeutic options [4,5]. Potential molecular mechanisms involve the inhibition of inward flow of calcium (Ca^2+^), reduction of neurotransmitter release, and inhibition of neuronal apoptosis [6,7,8,9,10]. CAA include three main classes: dihydropyridines, phenylalkylamines, and benzothiazepines [11]. Therefore, identifying novel CAA has become a critical issue that needs to be addressed.

Natural remedies, such as traditional Chinese medicines (TCM), represent an important source of new drug discovery [12]. Currently, a variety of screening models of natural active products have been developed based on phenotypes, modulated pathways, and corresponding molecular targets. However, the bioactivity identified using most screening studies is not closely related to the indications of the drug.

Shunaoxin pills (SNX), composed of Chuanxiong (root of *Ligusticum chuanxiong* Hort.) and Danggui [rhizoma of *Angelica sinensis* (Oliv.) Diels] is a proprietary Chinese medicine frequently used for the clinical treatment of chronic cerebral ischemia. SNX increases cerebral blood flow (CBF) in rats with cerebral ischemia [13], and its main components, Chuanxiong and Danggui, have many beneficial effects besides improving blood circulation, such as relief of headaches and anti-inflammatory effects [14]. Evidence suggests that SNX may contain novel CAA [15]. However, the specific active ingredients of SNX that antagonize Ca^2+^ are still unclear.

In this study, we developed an effective high-throughput screening system for the identification of the active ingredients of TCM based on their pharmacodynamic mechanisms. We used a transcriptomic approach to screen the underlying mechanism of SNX and then applied an activity-based ultra-high performance liquid chromatography with quadrupole time-of-flight mass spectrometry (UPLC/Q-TOF-MS/MS) assay to screen its active ingredients. We identified two active monomers that constitute the material basis of the beneficial effects exerted by SNX in CCH.

## 2 Materials and methods

### 2.1 Chemicals and reagents

SNX extract was provided by Tianjin Zhongxin Pharmaceutical Group Co., Ltd (Tianjin, China; batch number 677014). Ligustilide (Lig, 98%) was purchased from Tianjin YIFANG S&T Co., Ltd (Tianjin, China). Senkyunolide I (S I, 98%) was purchased from Macklin Biochemical Technology Co., Ltd (Shanghai, China). Nifedipine (Nif, 98%) was purchased from Solarbio Technology Co., Ltd (Beijing, China). Nicergoline tablets were purchased from Kunshan KRRP Pharmaceutical Co. Ltd (Jiangsu, China). The Ca^2+^ luciferase reporter plasmid pGL4.30 and Renilla luciferase reporter vector plasmid pRL-TK were purchased from Promega (Madison, WI, USA). All cell culture reagents were obtained from Gibco BRL Life Technologies (Carlsbad, CA, USA).

### 2.2 Animals

Sprague-Dawley rats (male, 250–300 g) were purchased from the Beijing Experimental Animal Center of Military Medical Sciences (Beijing, China). The rats were raised in a 12 h automatic light/dark cycle in a standard animal room with the temperature maintained at 25°C and had free access to water and food. All experimental protocols were performed in accordance with the Guide for the Care and Use of Laboratory Animals and approved by the Animal Ethics Review Committees of Tianjin University of Traditional Chinese Medicine (DW20181223-35).

### 2.3 Model of chronic constriction injury in rats and drug administration

Anesthesia with 10% chloral hydrate 0.3 mL/100 g was administered intraperitoneally, and the CCH model was developed by ligating the bilateral common carotid artery [16]. The rats were randomly divided into six groups (*n* = 6): the control (saline), model (saline), positive control (nicergoline, 2 mg/kg/day), and SNX (SNX 20, 10, and 5 mg/kg/day) groups. The treatment was administered intragastrically continuously for 8 weeks. After 8 weeks, the rats were anesthetized by intraperitoneal injection of 10% chloral hydrate (0.3 mL/100 g), lying prone, the head was shaved, the skin at the beginning was cut for about 2 cm, the exudate was removed with a cotton swab soaked in hydrogen peroxide, and the skull was exposed. The probe of Doppler flow meter was fixed on the skull with glue, and the rats were placed in supine position. The CBF of the rats was assessed using two-dimensional images obtained with the laser Doppler blood flow imaging system (Moor Instruments, Great Britain). Afterward, rats were decapitated after taking blood, and brain tissues were collected and divided into two parts. Half of the samples were preserved in formalin for hematoxylin–eosin (HE) staining, and the other were used for transcriptome analysis.

### 2.4 Transcriptome and bioinformatics analysis

Transcriptional analysis was undertook at the Beijing Genomics Institute in Shenzhen (BGI Tech). Brain samples from four groups (control, model, SNX high dose, and positive control) (*n* = 3) were selected for this analysis. The total RNA of the brain tissue samples was extracted, the original sequences were read and analyzed by Illumina HiSeq™ 2000 and compared with the reference genome to identify the differentially expressed genes. To this purpose, DESeq2 algorithm was employed. The *P*-value of each gene was adjusted using Benjamini and Hochberg’s approach to control the false discovery rate. Genes with a Log2Ratio of ≥1 and adjusted *P* ≤ 0.05, determined by DESeq, were classified as differentially expressed genes.

The differentially expressed genes were functionally annotated using gene ontology (GO) and pathway enrichment analysis. The intersection genes were selected and clustered using MeV software. The Kyoto Encyclopedia of Genes and Genomes (KEGG) (http://www.genome.jp/kegg/) was used to identify significant genes in the KEGG pathway database.

### 2.5 Sample preparation

Ten milligrams of extract of SNX were weighted and dissolved in 1 mL of methanol. The solution was vortexed for 1 min (min), ultrasonically dissolved for 30 min, and centrifuged at 4°C and 10,000×*g* for 15 min, and the resulting supernatant was used for UPLC analysis and cell culture after vacuum drying.

### 2.6 UPLC/Q-TOF-MS analysis

#### 2.6.1 UPLC separation

The samples were analyzed with a Waters ACQUITY UPLC system (Waters, Milford, USA), and the system was operated using MassLynx V4.1 software (Waters). A Waters ACQUITY BEH C18 column (1.7 µm, 2.1 mm × 100 mm; Waters) was used for sample separation. The injection volume was 4 µL. The column was maintained at 30°C and the flow rate was set at 0.4 mL/min. The optimal mobile phase consisted of a linear gradient system of (A) acetonitrile and (B) 0.1% formic acid in water: 0–10 min, 2–27% A; 10–18 min, 27–33% A; 18–20 min, 33–35.5% A; 20–22 min, 35.5–38% A; 22–24 min, 38–54% A; 24–26 min, 54–69% A; 26–28 min, 69–71% A; 28–30 min, 71–73% A; 30–32 min, 73–100% A; 32–33 min, 100% A; and 33–35 min, 100–2% A.

The resulting eluates were collected directly into a 96-well plate every minute. After collection, the 96-well plate was placed in a vacuum drying oven and dried under reduced pressure at 50°C. After the solvent was completely evaporated, 100 µL of cell culture medium was added to each well. The complete medium was placed on a shaker for 20 min and then sonicated at 37°C for 30 min to dissolve it completely. Finally, the solution was used in subsequent experiments.

#### 2.6.2 Mass spectrum analysis

Accurate mass and MS/MS measurements were obtained using a detector with a dual electrospray ionization (ESI) probe and a Micromass Q-TOF micro Synapt High-Definition Mass Spectrometer (Waters, Milford, USA). The mass spectra were acquired in positive ionization modes (ESI^+^), and the optimal conditions of analysis were set as follows: the source temperature 110°C; the temperature and flow of the desolvation gas 350°C and 600 L/h, respectively; the capillary voltage 2.5 kV for the negative mode; the sampling cone voltage 30 V; the capillary voltage 3.0 kV for the positive mode; extraction cone voltage 45 V; cone gas flow 50 L/h; and collision energy 6.0 eV. All analyses were performed using the LockSpray interface to ensure accuracy and reproducibility. The Q-TOF Premier sample collection was 0.1 s with a 0.02 s scan interval delay, and the first resolving quadrupole was performed in the wide-pass mode (100–1,500 Da). Leucine encephalin amide acetate (200 pg/µL) was used as the lock mass ([M+H]^+^ = 555.2931, [M−H]^−^ = 553.2775) with a flow rate of 20 µL/min.

### 2.7 Cell culture

Human embryonic kidney 293T (HEK 293T) cells were obtained from the American Type Culture Collection (Manassas, VA, USA). 293T cells were cultured in Dulbecco's Modified Eagle’s Medium supplemented with 10% fetal bovine serum, 100 U/mL penicillin, and 0.1 mg/mL streptomycin in a humidified incubator with a 5% CO_2_ supply at 37°C.

### 2.8 Luciferase reporter assay for calcium antagonism

HEK 293T cells were co-transfected with 100 ng/well luciferase reporter plasmid PGL4.30 (Promega) and 10 ng/well Renilla luciferase reporter vector pRL-TK plasmid (Promega) [17]. After 24 h co-transfection, 293T cells were pretreated with Nif (10 µM), fractions of SNX, Lig (0.5, 5, and 50 µM), and S I (0.5, 5, and 50 µM) for 6 h. Then, except for the control group, the model group and the administration group were stimulated with ionomycin (1 mmol/L) and 12-myristates-13-acetate (1 mg/mL) for 6 h. Cells were lysed after discarding the cell supernatant and luciferase activity was detected using the Luciferase Reporter Assay System (Promega). The relative activities of firefly luciferase and Renilla luciferase were used to normalize differences in transfection efficiency.

### 2.9 Statistical analysis

All data are expressed as mean ± standard deviation (SD). Differences between two groups were analyzed by *t*-test. Multiple group comparisons were made using the analysis of variance test. Statistical tests were performed using statistical analysis software (Graph Pad Prism verion7.0; Graph Pad Software, Inc., San Diego, CA, USA). Statistical significance was set at *P* < 0.05.

## 3 Results

### 3.1 SNX induces cerebrovascular dilatation and protects against CCH-induced nerve cell injury in rats

**Figure 1:**
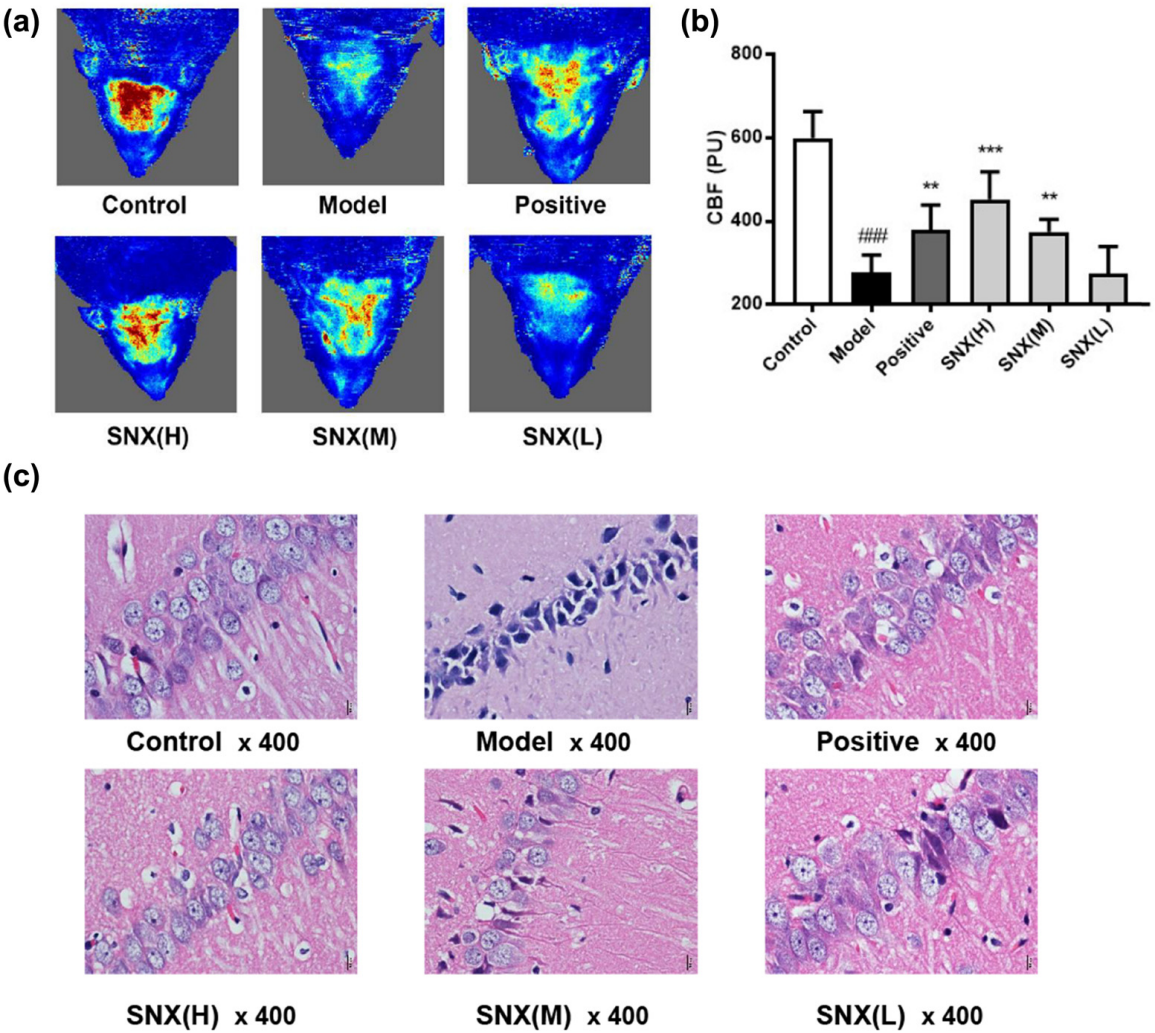
SNX-induced cerebrovascular dilation in CCH rats. (a) The effect of SNX on CBF in CCH rats assessed using a Doppler blood flow imaging system. (b) Gray analysis of CBF images. (c) Images of hippocampal neurons of rats with HE staining (400-fold); **P < 0.01, ***P < 0.001 versus the model group and ^###^ *P* < 0.001 versus the control group (*n* = 6).

Vasodilation is the most desired outcome of an effective treatment for CCH, as it increases the blood flow. A Doppler blood flow assessment imaging scanning system was used to evaluate the effect of SNX on CBF in rats. The results showed that the CBF of rats decreased significantly after induction of the injury. CBF was significantly improved in the positive control and the high- and medium-dose SNX groups when compared to the model group (Figure 1a and b, **P < 0.01, ***P < 0.001).

CCH leads to a series of damaging changes in the hippocampal CA1 region [18]. In this study, compared to those in the control group, the nerve cells in model rats were indistinctly visible in the nucleus, and plasma was seldom observed in the corresponding HE-staining sections (Figure 1c). However, the treatment with high and medium doses of SNX and the positive control protected partly the integrity of nerve cells and inhibited the proliferation of glial cells, indicating that SNX could prevent CCH-induced nerve cell injury.

### 3.2 Calcium homeostasis regulation was the main mechanism underlying the effect of SNX against cerebral ischemia

**Figure 2:**
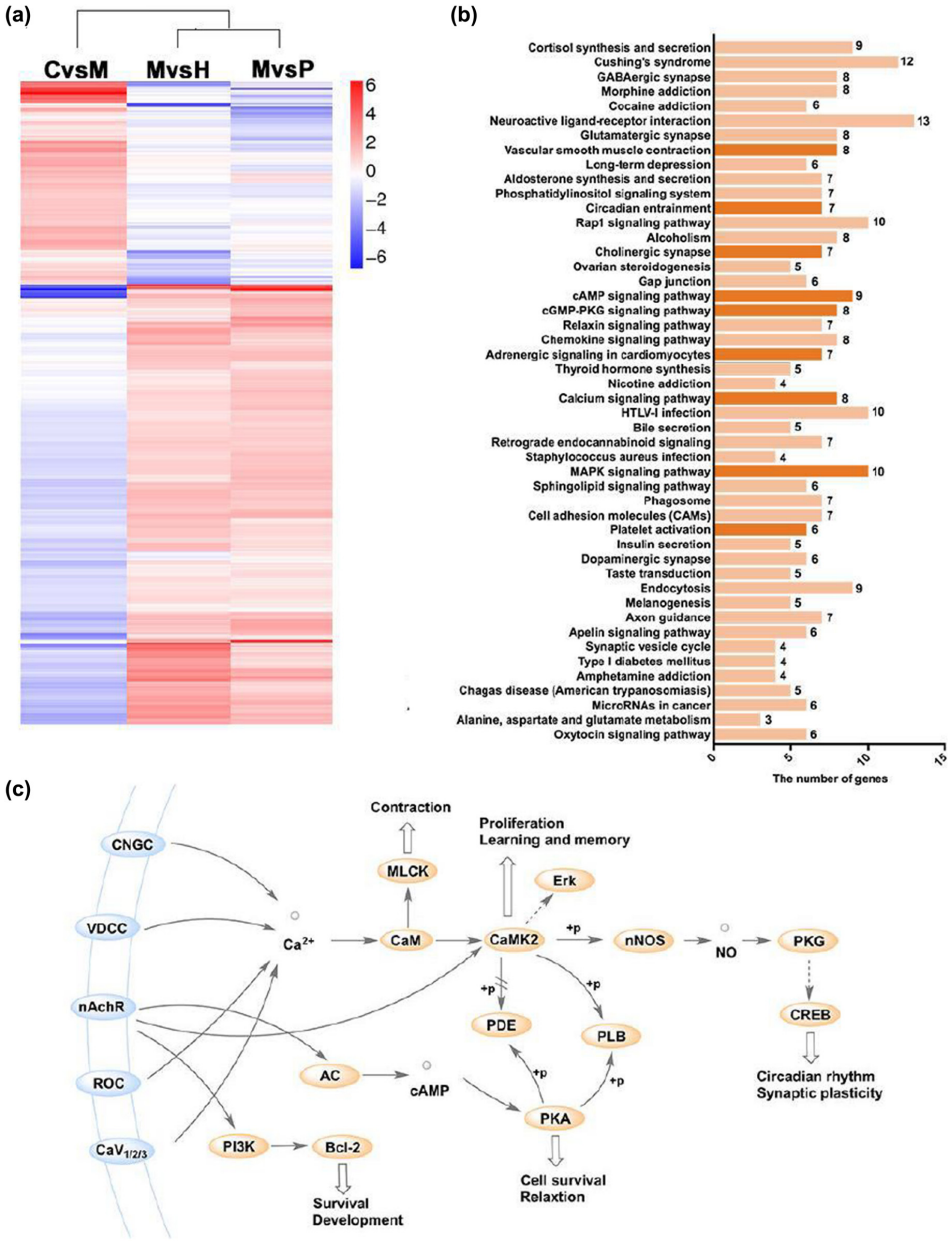
The RNA-Seq transcriptional analysis of brain tissues. (a) Heatmap plot of 306 differentially expressed genes. The high dose of SNX and nicergoline regulated the genes expressed differentially owing to surgical injury. Red, upregulation; blue, downregulation. (b) Signaling pathway annotation of differentially expressed genes for the top 48 related signaling pathways. The nine calcium-related signaling pathways are represented with dark color. (c) Bioinformatic analysis – prediction of the potential functions of SNX associated with the calcium homeostasis regulation-related signaling pathways.

**Figure A1:**
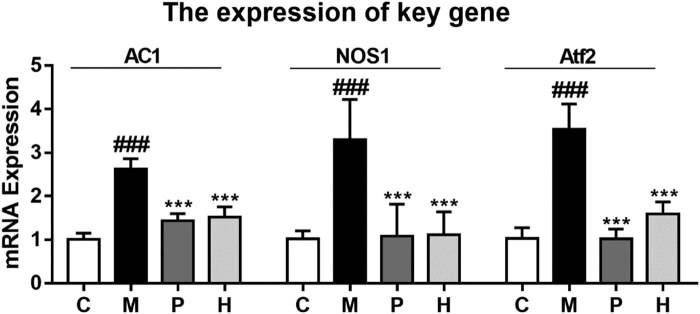
Expression of AC1 mRNA, NOS1 and Atf2-mRNA in different groups. C, control; M, model; P, positive; H, the high-dose of SNX (H). **P < 0.01, ***P < 0.001 versus the model group and ^*###*^ *P* < 0.001 versus the control group (*n* = 6).

To explore the mechanisms underlying the protective effect of SNX against cerebral ischemia, RNA-Seq transcriptome analysis was used to compare changes in the level of gene expression in the brain tissue before and after administration. An absolute value of log2Ratio greater than or equal to 1 was set as the threshold to identify the genes differentially expressed between groups. Applying this criterion, 306 differentially expressed genes were identified between the three groups (control vs model, model vs high-dose of SNX, and model vs positive control). Sixty-six differentially expressed genes were upregulated and 240 genes were downregulated in the model group when compared to the control. In total, 227 differentially expressed genes were upregulated and 55 genes were downregulated in the high-dose SNX group when compared to the model group. Log2Ratio values were used for cluster analysis using MeV software, and the upregulated and downregulated differentially expressed genes are shown in red and blue, respectively, in Figure 2a. In addition, we used real-time PCR to verify the key genes of AC1, NOS1, and Atf2 identified by RNA-Seq, and the results were consistent with those of genomics (Figure A1). These differentially expressed genes were primarily enriched in 48 KEGG pathways, including 9 calcium-related signaling pathways (vascular smooth muscle contraction, circadian entrainment, cholinergic synapse, cAMP signaling pathway, cGMP-PKG signaling pathway, adrenergic signaling in cardiomyocytes, calcium signaling pathway, MAPK signaling pathway, and platelet activation) (Figure 2b). These results indicate that the regulation of intracellular calcium homeostasis is one of the core mechanisms underlying the effects of SNX against cerebral ischemia. Integrating the information on the nine signaling pathways, as shown in Figure 2c, we reveal that, by antagonizing calcium, SNX might play a role in muscle contraction, cell proliferation, learning and memory, suppression of apoptosis, survival enhancement, and circadian plasticity.

### 3.3 Screening of components in SNX with CAA properties

**Figure 3:**
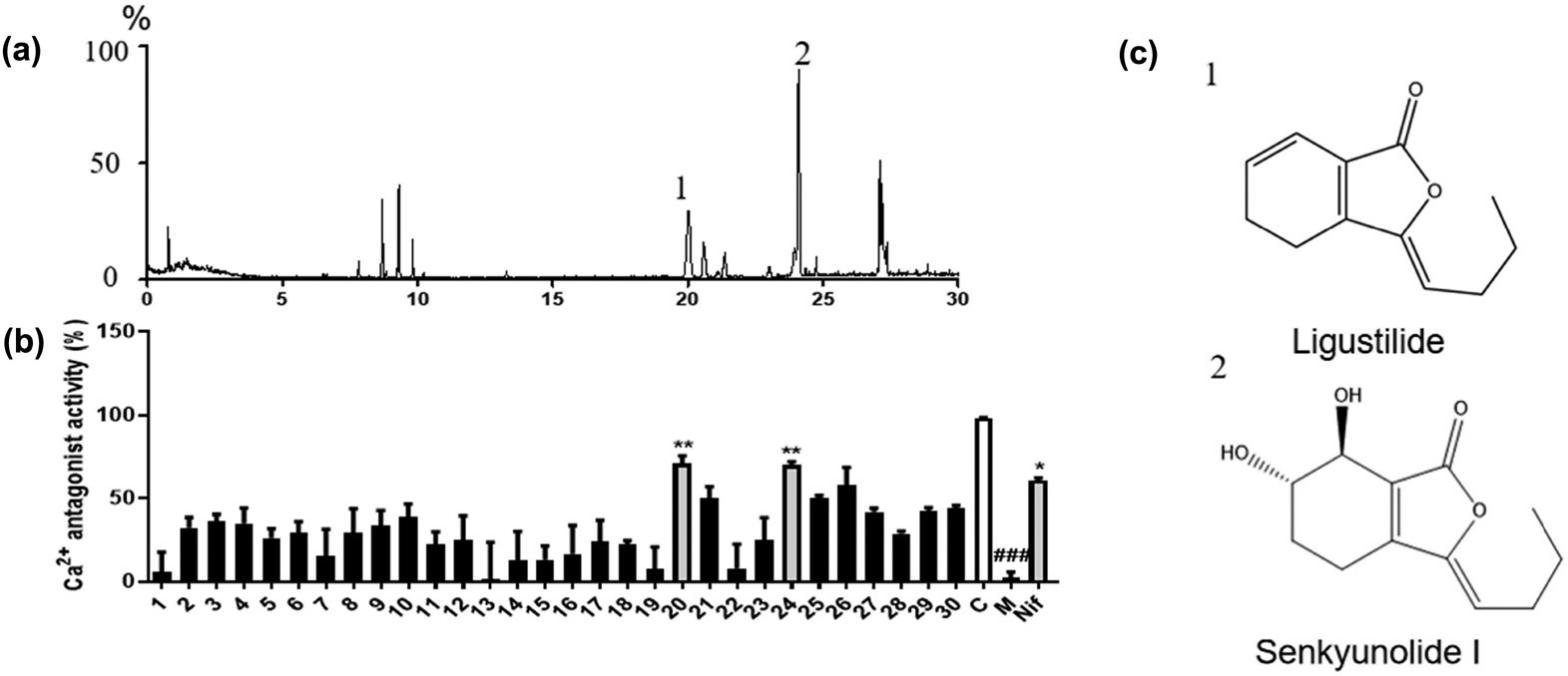
Screening of SNX components that exert calcium antagonistic activity. (a) Identification of SNX components using UPLC/Q-TOF-MS/MS. (b) Dual-luciferase reporter assay for calcium antagonistic activity of each component in SNX. C, control; M, model. (c) Chemical structure of calcium antagonistic components in SNX. Data are shown as the mean ± SD; *n* = 3 per group. ^##^ *P* < 0.01 compared to the control group; *P < 0.05, **P < 0.01 compared to the model group.

To identify the active pharmacological ingredients with CAA properties contained in SNX, we further employed a screening system integrating UPLC/Q-TOF-MS/MS and dual-luciferase reporter calcium assay. As shown in Figure 3a, most of the chromatographic peaks were completely separated after the UPLC/Q-TOF-MS/MS. After UPLC separation, the calcium inhibitory effect of each fraction was tested. As shown in Figure 3b, two fractions (numbers 1 and 2) showed significant inhibitory activities against Ca^2+^, and these were identified as Lig and S I.

### 3.4 Demonstration of the calcium antagonistic effect of Lig and S I

**Figure 4:**
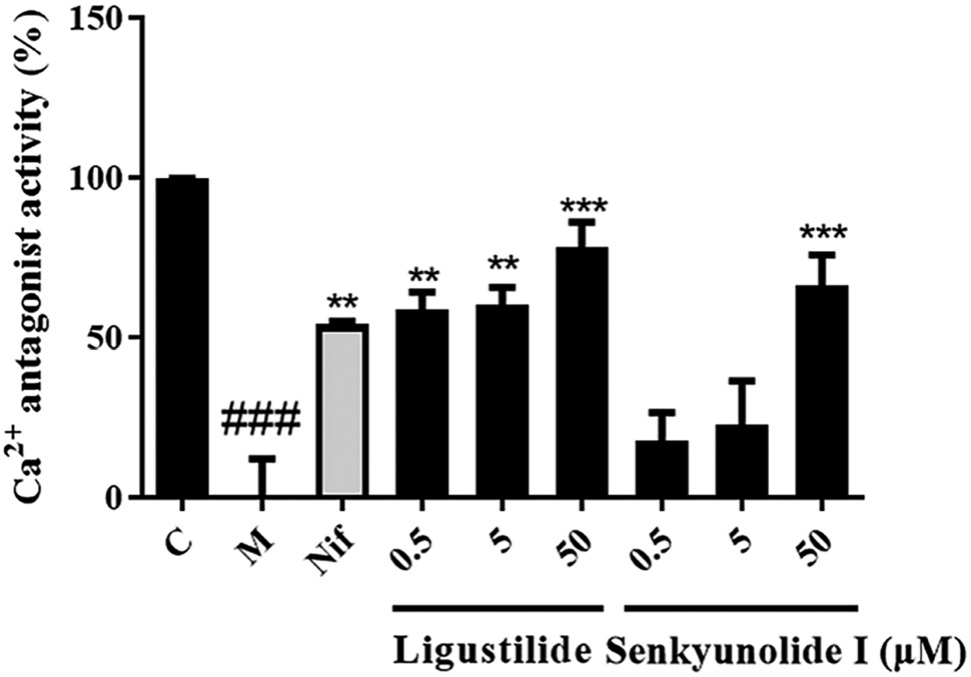
Dual-luciferase reporter assay to study the calcium antagonistic effect of different concentrations of Lig and S I. Data are shown as the mean ± SD; *n* = 5 per group. ^###^ *P* < 0.001 compared with the control group; **P < 0.01, ***P < 0.001 compared to the model group; C, control; M, model .

To confirm the accuracy of the activity-based screening system, the calcium antagonistic activity of Lig and S I was evaluated using standards of these active compounds and a dual-luciferase reporter assay. As shown in Figure 4, compared with the model group, nifedipine (5 µM), Lig (0.5, 5, and 50 µM), and S I (50 µM) significantly inhibited the increase in intracellular calcium induced by ionomycin and PMA. These results further confirmed that Lig and S I are the main pharmacoactive components of SNX that exert calcium antagonistic properties.

## 4 Discussion

TCM is an important source of active natural products. Currently, one of the key problems to be solved is the quick and effective screening of active natural ingredients contained in the medicinal products used in TCM [19]. The current methods for identifying these active ingredients employ phenotypic, signaling pathway, and molecular target-based screening [20,21,22]. However, they all have advantages and disadvantages. The phenotype-based screening method helps to understand the effect of active ingredients on the overall macro level, such as the entire animal organism; however, it does not take into consideration micro factors, such as target proteins and specific mechanisms. Target- and signaling pathway-based screening methods can determine the activity of drugs on molecular targets with high specificity and sensitivity; however, it is difficult to determine whether medicines used by TCM contain active ingredients that act precisely on these targets and pathways. Combining phenotypic, target-, and signaling pathway-based screening may solve this problem.

In the present study, we developed a CCH model by ligating bilateral common carotid arteries in rats. We used this model to demonstrate that SNX dilates cerebral blood vessels and increases CBF. Then, we analyzed the changes in the levels of gene expression in rat brain tissue between treatment, model, and control groups using the transcriptome analysis and found that calcium-related signaling pathway is highly modulated by treatment with SNX. Furthermore, one of the core mechanisms underlying the beneficial effects of SNX against CCH is the regulation of calcium homeostasis. An increase in intracellular free calcium concentration causes vascular smooth muscle contraction and spasm [23,24]. CAA can inhibit the entry of Ca^2+^ into the cytosol, reduce spasm and subsequently, and alleviate CCH [25,26].

Therefore, we developed a rapid screening system for identifying CAA. This system is based on the separation of the major active ingredients in SNX using UPLC/Q-TOF-MS/MS and further screening of its pharmacodynamic components using a dual-luciferase reporter calcium assay. Two CAA, Lig and S I, were identified, which is consistent with previously reported data. The calcium antagonistic effects of senkyunolide A and ferulic acid in SNX have also been reported [17,27,28]. However, we did not find them in our screening, which may be related to their low content in SNX.

In addition, genomic studies indicate that the anti-inflammatory effect might be a main contributor to the effect of SNX against CCH. This represents one of the directions of our further research: to screen the anti-inflammatory active components contained in SNX.

## 5 Conclusion

We established a rapid screening method suitable for the discovery of active natural ingredients contained in TCM by integrating genomics and target pathway-oriented spectroscopic analysis. This provides a more complete screening system for key pharmacodynamic active ingredients contained in Chinese herbal compounds based on their clinical activity.

## Quantitative real-time PCR

Six rats’ brain tissues of sampling from each group were used for the expression of key gene detection. RNA extraction accorded to the instruction of RNA extraction kit (ThermoFisher Scientific, Waltham, MA, USA). The cDNA was synthesized using a High-Capacity cDNA Reverse Transcription Kit (ThermoFisher Scientific, Waltham, MA, USA). PCR amplification was performed and analyzed using the GoTaqqPCR Master Mix (Promega), and the emission intensity from the BRYT Green was detected with an iCycler detection system (Bio-Rad, Hercules, CA, USA). Select Rat β-actin as inner reference to compute the expression of Rat calcium-activated adenylate cyclases (AC1) mRNA, nitric oxide synthase (NOS1) mRNA and activating Transcription Factor 2 (Atf2) mRNA (Figure A1). The PCR primers were designed and shown in Table A1.

**Table A1:** Primers and probes for real-time PCR

Gene	Primers sequence
Rat AC1	Forward-5′-TGAGCTTCGTCCTGTGACGATCGTGTTTG-3′
	Reverse-5′-TGTGCACACAGGCAGCTTGGAT GG-3′
Rat NOS1	Forward-5′-GGAATATGAGGAGAAGTGTAG-3′
	Reverse-5′-CTGAGTGAGGAGTGTAG-3′
Rat Atf2	Forward-5′-CAGTCAGAAGAGTCTCGTCCACA-3′
	Reverse-5′-GAAGCTGCTGCTCTATTTCGTTC-3′
Rat β-actin	Forward-5′-CACGATGGAGGGGCCGGACTCATC-3′
	Reverse-5′-TAAAGACCTCTATGCCAACACAGT-3′
